# Papillary microcarcinomas of the thyroid gland and immunohistochemical analysis of expression of p53 protein in papillary microcarcinomas

**DOI:** 10.1186/1479-5876-4-28

**Published:** 2006-07-05

**Authors:** Demet Corapcioglu, Serpil D Sak, Tuncay Delibasi, Vedia Tonyukuk, Nuri Kamel, Ali R Uysal, Savas Kocak, Semih Aydintug, Gurbuz Erdogan

**Affiliations:** 1 Department of Endocrinology and Metabolism, Ankara University School of Medicine, Ankara, Turkey; 2 Department of Pathology, Ankara University School of Medicine, Ankara, Turkey; 3 Department of Surgery, Ankara University School of Medicine, Ankara, Turkey

## Abstract

**Background:**

Thyroid papillary microcarcinoma (TPM) is defined according to WHO criteria as a thyroid tumor smaller than 1–1.5 cm. TPMs are encountered in 0.5–35.6 % of autopsies or surgical specimens where carcinoma had been unsuspected. The purpose of the present study was to evaluate patients who had TPMs in terms of clinical findings, histopathological features and immunohistochemical evidence of expression of the tumor suppressor gene p53.

**Methods:**

A total of 44 patients with TPMs less than 1.0 cm in diameter were included in the study. The patients were evaluated clinically and the tumors were evaluated in terms of their histopathological and immunohistochemical features, including expression of p53.

**Results:**

The female/male ratio was 2.8/1, and the median age at time of diagnosis was 49 years (range 20–71 years). The maximum diameter of the smallest focus was 0.1 mm, and that of the largest was 10 mm microscopically. The mean diameter of all tumors was 5.7 mm. There was no correlation between tumor size and age or gender. Of the TPMs, 72 % were found in the right lobe, 24 % in the left lobe and 4 % in the isthmus. Fine-needle aspiration biopsy provided the diagnosis of TPM in only 43.2 % of the patients. All patients were treated with surgery, with 20 undergoing conservative surgery, i.e. lobectomy or isthmusectomy, and 24 undergoing total thyroidectomy. Frozen section provided the diagnosis of TPM in only 56.8 % of the patients. We found lymphocytic thyroiditis in 13.6% of patients, follicular variants in 11.9%, capsular invasion in 26.8%, lymph node involvement in 11.9%, soft tissue metastases in the neck in 12.1% and multifocality in 31.7 %, and none of these were related to age or gender (p > 0.05). No distant metastases were observed during approximately 10 years of follow up. We found p53 positivity in 34.5 % of TPM tumors. However, p53 expression was not statistically related to age or gender.

**Conclusion:**

Our findings imply that TPMs may not be entirely innocent since they are associated with signs of poor prognosis such as capsular invasion, multifocal presentation, lymph node involvement and p53 positivity. Therefore, TPMs should be evaluated and followed like classical papillary cancers.

## Background

Thyroid papillary microcarcinoma (TPM), also known as occult papillary carcinoma (OPC), is usually defined as a thyroid tumor smaller than 1–1.5 cm [1,5]. The term "occult thyroid papillary carcinoma" had been used before the WHO proposed a major diameter of 1 cm for occult papillary carcinoma, and renamed it as microcarcinoma [6]. Despite this proposal, different terms are currently used to define this thyroid cancer such as small, tiny, minute, minimal or incidental thyroid papillary cancer [7,10].

TPMs are found at rates of 0.5–35.6 % at autopsy or in surgical specimens where carcinoma had been unsuspected [11,18]. Some authors suggest that small tumors are very unlikely to be clinically significant even though up to 60 % of these lesions are associated with lymph node metastases which may be their presenting features [19,20]. TPMs that either have lymph node metastases or are multifocal with extracapsular invasion may be associated with lung metastases. Such cases are rare but they have significant morbidity and mortality [20,21]. Other authors take it as widely accepted that the recurrence and mortality risks of microcarcinomas are low [22,24].

Given these somewhat mixed views regarding the clinical importance of TPMs, it seems desirable to study these tumors in detail, particularly in terms of any biochemical markers they exhibit. One such marker is the protein product of p53, a tumor suppressor gene. Mutations in or deletions of the p53 gene are among the most frequently detected genetic changes in human cancers [25,26]. The gene itself is located on the short arm of chromosome 17 [26]. The p53 protein is capable of inhibiting cell proliferation and transformation [27,28]. Several studies have shown a correlation between mutated p53 gene expression and degree of tumor differentiation [29,30]. It has also been reported that p53 gene expression is an indicator of poor prognosis, and might be important in the future for planning the treatment of TPMs [31,32].

The purpose of the present study was to evaluate patients with TPMs in terms of clinical findings, histopathological features and immunohistochemical evidence of p53 expression, and to describe the relations between these.

### Patients and methods

We reviewed the clinical records of 255 consecutive patients who underwent surgery for thyroid cancer between 1994 and 2002 at Ankara University Hospital in Ankara, Turkey. Of these patients, 44 had tumors that were less than 1.0 cm in diameter, and these tumors were classified as microcarcinoma. Of these 44 patients, 29 had pathological preparations that we were able to stain for p53 expression. The specimens of the 15 other patients could not be stained for various reasons, including inadequate pathological slides. This retrospective study was performed in accordance with the ethical requirements of our institution. All histopathologic and cytologic materials were reviewed by the same pathologist and were evaluated according to WHO recommendations [6].

The variables examined in this study included age and gender of the patient, tumor size, histological features (multifocality, invasion of the thyroid capsule, etc.), the presence of distant or regional metastases and the expression of p53 protein.

To detect p53 gene expression immunohistochemically, we used a monoclonal antibody that reacts with both mutant and wild type p53 protein (clone DO7+BP53-12, Neomarkers, CA, USA). Sections 4–6 μm thick were cut from tissue fixed with 10 % buffered formalin, embedded in paraffin blocks and treated with xylene for deparaffinization and were rehydrated in decreasing alcohol concentrations. Antigen retrieval was performed by heating samples in citrate buffer (pH 7.0) in a pressure cooker. Endogenous peroxidase activity was blocked by H_2_O_2. _A streptavidin biotin horseradish peroxidase system with a computerized staining apparatus (Ventana, Nexus) was used for staining procedures. Antibody dilution was 1:100. Diaminobenzidine (DAB) was used as a chromogen and counterstaining was performed with hematoxylin. A larynx carcinoma with known p53 expression served as the positive control. For the negative control, the procedures were the same but PBS was substituted for the primary antibody. The slides were evaluated by light microscopy, and brown nuclear staining was accepted as positive. The most extensively stained area was examined and the percentage of positively stained tumor cells was estimated.

Statistical analyses were performed with SPSS v.11.0 software (SPSS Inc., Chicago, USA).

## Results

Among the 44 patients in the study, the female to male ratio was 2.8/1, and the median age at time of diagnosis was 49 years (range 20–71 years). Mean follow-up period of the patients was approximately 10 years. None of the patients had previously received radiation therapy directed at cervical lymph nodes and none had a family history of thyroid cancer.

The maximum diameter of the smallest focus was 0.1 mm, and that of the largest was 10 mm microscopically. The mean diameter of all tumors was 5.7 mm, and the mean diameters in males and females were 5.4 mm and 5.8 mm, respectively. When the patients were classified as younger or older than 50 years, the mean diameter of TPMs was 5.5 mm in the younger patients and 5.8 mm in the older ones. Tumor size was not related to age or gender (p > 0.05). Of the TPM foci, 72 % were found in the right lobe, 24 % in the left lobe and 4 % in the isthmus.

Table 1 compares fine needle aspiration biopsy (FNAB) and frozen section in terms of detectability for TPM in our patients. FNAB provided the diagnosis of papillary carcinoma in only 43.2 % of the TPM tumors. All patients underwent surgery. Conservative methods, i.e. lobectomy and isthmusectomy, were used in 20 patients and total thyroidectomy was performed in 24 patients. Frozen section studies provided the diagnosis of TPM in only 56.8 % of the patients that were examined by frozen section. Of the patients with malignancy demonstrated by FNAB, 6.8 % were diagnosed with benign disease on frozen section.

**Table 1:** Comparison of FNAB and frozen section in terms of their detection of TPM

	FNAB (% of patients)	Frozen section (% of patients)	Postoperative pathology (% of patients)
TPM	43.2	56.8	100
Follicular variant	0	0	11.9

Following surgery, the 24 patients who had bilateral total thyroidectomy received I^131 ^ablation therapy. The dosage of I^131 ^ranged from 75 to 200 mCi. Thyroid hormone replacement therapy (LT4) was continuously administered to these patients as well as to the 20 patients who had received lobectomy or isthmusectomy. The dosage of LT4 ranged from 100 to 175 μg.

Table 2 shows the histopathologic and other features of the tumors. Due to their small size most TPMs were not detected grossly. All tumors exhibited the characteristic features of papillary carcinoma such as ground-glass nuclei with nuclear grooves and occasional nuclear inclusions. We found lymphocytic thyroiditis in 13.6% of patients, follicular variants in 11.9%, capsular invasion in 26.8%, lymph node involvement in 11.9%, soft tissue metastases in the neck in 12.1% and multifocality in 31.7 %, and none of these were related to age or gender (p > 0.05). During approximately 10 years of follow-up, no distant metastases were observed and there were no deaths.

**Table 2:** Histopathologic and other features of the tumors in this study

	(n)	%
Lymphocytic thyroiditis	6(44)	13.6
Follicular variant	5(42)	11.9
Capsular invasion	11(41)	26.8
Lymph node involvement	5(42)	11.9
Soft tissue metastases in the neck	5(41)	12.1
Distant metastases	0	0
Multifocality	13(41)	31.7
p53 positivity	10(29)	34.5

We found p53 positivity in 34.5 % of the TPM tumors. There was no correlation between p53 expression and sex, age, histological variant of tumors, capsular invasion, multifocality or presence of local or regional lymph node metastases (p > 0.05) (Table 2).

## Discussion

When we examine the cases of TPM in our patients and in the literature, we find that despite their small size, TPMs are not biologically innocent in all cases and may show capsular invasion, multifocality, regional lymph node metastases and p53 protein positivity at the time of diagnosis. These features associated with a portion of TPMs are the poor prognostic factors for differentiated thyroid malignancies (DTC). TPMs might therefore need to be evaluated and followed in the same way as classical thyroid papillary carcinomas.

Among our patients, females appeared to be more frequently affected, as is the case in other types of papillary carcinoma. Our female to male ratio was 2.8/1, similar to that in Salvadori's microcarcinoma study [34]. Kasai and Sakamoto have reported an even higher female to male ratio of 9/1 in their series [7]. In contrast, some authors reported that OPC was more common in men than in women, although non-occult papillary carcinoma was more common in women [12,35]. These latter findings favor the view that OPC is a different clinical entity and not the early stage of non-occult papillary carcinoma.

The median age at time of diagnosis of TPM was 49 years in our patients. Our patients' ages ranged from 20 to 71, a wide range similar to those previously reported in the literature [14,15].

With fine needle aspiration biopsy (FNAB) of the thyroid we were able to detect malignancy in only 43.8 % of our TPM tumors. This ratio is slightly lower than some previously reported rates, which range from 35.8 % to 55.5 % [36,41,46]. The reason for our lower rate of carcinoma detection with FNAB may be the smaller sizes of the tumors we have examined. Tumors with diameters less than 10 mm have been evaluated in our present study whereas tumors up to 15 mm in diameter have been included in most of the previous studies. Still, our rate is more or less close to the 49.3 % rate of carcinoma detection in OPC with ultrasound-guided FNAB reported by Jen Der Lin et al [36]. The rate of detection of carcinoma in frozen section examination is also low (57 %) in our study. Small tumor size might be responsible for this as well. In the study by Jen Der Lin et al., malignancy was detected in 73 % of the OPC cases on frozen section examination [36].

With regard to pathological features, capsular invasion was observed in 26.8 % of our patients. Salvadori et al. reported an incidence of 15.2 % of capsular invasion in a series that included tumors larger than those evaluated in our study [34]. Furmanckuk et al. reported that one patient out of 10 with papillary or mixed papillary-follicular OPC had capsular invasion [16]. Harach et al. reported a 17 % rate of capsular invasion in their series [12]. They observed a statistically significant association between tumor size and capsular invasion, larger tumors being more likely to invade the capsule [12].

The occurrence of multiple foci of carcinoma in OPC is thought to be due to intrathyroidal tumoral invasion through lymphatics rather than neoplastic transformation of multiple clones of cells located in various parts of the thyroid [37]. Multiple carcinomatous foci can sometimes be observed in both lobes of the thyroid [3,12,14,15]. Multiple foci of papillary carcinoma were identified in 31.7 % of TPM tumors in our series. The incidence of multifocality in OPC has been reported as 35.6% by Harach [12], 30.4 % by Yamamoto [14], 20.6 % by Pelizzo [15], 31.5 % by Furmanckuk [16], and 23.6 % by Salvadori [34]. Hazard has noted that multifocality is associated with metastases [38]. A small tumor can therefore be multifocal and carry the risk of metastasis. No relationship has been reported, so far, between the multiplicity of tumors and age or gender, and we found no such relation in our patients.

We found lymphocytic infiltration within the tumor stroma or around the tumor foci in six patients (13.6 % of TPM tumors). Harach et al. have reported a rate of 27.7 % for lymphocytic reaction within the tumor stroma or around the invading tumor islands [12]. Such lymphocytic infiltration was associated with invasive OPC and larger tumors, and it was thought to represent host reaction to tumor. The occurrence of lymphocytic infiltration limited to tumor periphery therefore seems unrelated to lymphocytic thyroiditis [12]. However, Hirabayashi and Lindsay have reported a higher than expected frequency of non-occult papillary carcinoma in patients with thyroiditis [39], and it has also been reported that the presence of lymphocytic thyroiditis around tumors indicates a good prognosis [40].

Lymph node metastases are characteristic features of all types of papillary carcinoma including OPC. However, they are of doubtful clinical significance. They are thought to indicate early recurrence and poor prognosis by some authors [31,42]. On the other hand, other investigators report that regional lymph node metastases do not affect survival [43,45]. Regional lymph node metastases were detected in 11.9 % of our patients and this is an incidence that is well within previously reported ranges. Frequencies of lymph node metastases in OPC have been reported as zero by Pelizzo et al. [15], as 0.3 % by Yamamoto et al [14], as 23 % by Hazard et al. [38], as 39 % by Woolner et al. [41], as 40 % by Hubert et al. [10], and as 42 % by Kasai et al. [7]. The highest reported frequency of lymph metastases is 70.8 % in the study by Salvadori et al. on OPC patients with tumor sizes less than 15 mm [34].

The presence of distant metastases indicates a poor prognosis for OPC [31]. Although distant metastases are rare in OPC as deduced from follow-up [34] or from findings in autopsy series [14-16], metastases to lung, trachea, pleura, vertebral column, pericardium, cerebrum, femur, humerus and mediastinum have been reported [1,46,53]. The smallest reported tumor with distant metastases was 3 mm in diameter [46]. No distant metastases were present in our patients with TPM during a follow-up period of approximately 10 years.

Similar to previous reports [14,15] tumors were located in the right lobe in 72 % of our patients, in the left lobe in 24 %, and in the isthmus in 4 %. Although the significance of this pattern of uneven distribution has not yet been clarified, nodules located in the right thyroid lobe may deserve a more detailed evaluation.

Tumor size has been reported as an important prognostic factor in papillary and follicular carcinomas of the thyroid [31]. In two autopsy series the mean diameter of occult thyroid carcinoma was 1.7 mm and 1.98 mm, respectively [14,16]. Tumor diameters in our series ranged from 1 to 10 mm (mean 5.7 mm). No association was found between tumor size and age or gender in those autopsy series [14,16]. The same was true in our series. One of the larger recent studies reported that approximately one in four patients with a papillary thyroid cancer no more than 1.5 cm in size developed persisting or relapsing disease after surgery [62].

Over-expression of p53 and mutations in the p53 gene are more frequent in poorly differentiated and anaplastic carcinomas than in well-differentiated papillary and follicular carcinomas [32,54,63]. Also, p53 gene expression is reported to be a significant and independent prognostic factor for cause-specific and crude survival in these patients [31,32]. Anaplastic changes following insufficient radioiodine therapy have been shown to be associated with p53 gene mutation in differentiated thyroid carcinoma [64]. There is a higher rate of p53 gene mutation in the tall cell variant of papillary thyroid carcinoma than in the common variant [65]. Immunohistochemical detection of p53 protein by means of prolonged half-life is thought to be associated with the occurrence of p53 gene mutations since the prolongation of the protein's half-life has been regarded as a direct consequence of structural changes attributable to missense point mutations [25]. More recent investigations have demonstrated that wild type p53 can be stable up to approximately 24 hours [33]. The mutated p53 protein can remain present in tissues for longer than 24 hours whereas the normal protein cannot. Of our patients, only 34.5 % tested positive for p53 and this was not significantly associated with age, gender, capsular invasion, lymph node metastases, multifocality, tumor location, histologic variant of tumor, or tumor diameter.

## Conclusion

Our findings in this study imply that TPMs may not be innocent since they can be associated with indicators of poor prognosis such as capsular invasion, multifocal presentation, lymph node involvement and p53 positivity. Therefore, thyroid papillary microcarcinomas should be evaluated and followed like other papillary cancers. In order to reach a clearer understanding of the relationships between p53 positivity and TPM, p53 gene mutations should be detected by using PCR methods, and clinical outcomes of these mutations should be evaluated in longitudinal studies in the same patients.

**Table 3:** Persentages in p53 positive and p53 negative patients according to histopathologic features.

	p53 positive n (%)	p53 negative n (%)	Total n
Lymphocytic thyroiditis	2 (33.3)	4 (66.7)	6
Follicular variant	2 (40.0)	3 (60.0)	5
Capsular invasion	5 (45.5)	3 (54.5)	11
Lymph node involvement	3 (60.0)	2 (40.0)	5
Soft tissue metastases in the neck	4 (66.7)	2 (33.3)	5
Multifocality	7 (53.8)	6 (46.2)	13
